# PollenCALC: Software for estimation of pollen compatibility of self-incompatible allo- and autotetraploid species

**DOI:** 10.1186/1471-2105-13-125

**Published:** 2012-06-07

**Authors:** Andrea Arias Aguirre, Bernd Wollenweber, Ursula K Frei, Thomas Lübberstedt

**Affiliations:** 1 Department of Agronomy, Iowa State University, Agronomy Hall, Ames, IA, USA; 2 Department of Agroecology, Faculty of Science and Technology, Aarhus University, Aarhus, Denmark

**Keywords:** Disomic inheritance, Polyploidy, Self-incompatibility, Software, Tetrasomic inheritance

## Abstract

**Background:**

Self-incompatibility (SI) is a biological mechanism to avoid inbreeding in allogamous plants. In grasses, this mechanism is controlled by a two-locus system (S-Z). Calculation of male and female gamete frequencies is complex for tetraploid species. We are not aware of any software available for predicting pollen haplotype frequencies and pollen compatibility in tetraploid species.

**Results:**

PollenCALC is a software tool written in C++ programming language that can predict pollen compatibility percentages for polyploid species with a two-locus (S, Z) self-incompatibility system. The program predicts pollen genotypes and frequencies based on defined meiotic parameters for allo- or autotetraploid species with a gametophytic S-Z SI system. These predictions can be used to obtain expected values for for diploid and for (allo- or autotetraploidy SI grasses.

**Conclusion:**

The information provided by this calculator can be used to predict compatibility of pair-crosses in plant breeding applications, to analyze segregation distortion for S and Z genes, as well as linked markers in mapping populations, hypothesis testing of the number of S and Z alleles in a pair cross, and the underlying genetic model.

## Background

Self-incompatibility (SI) is a biological mechanism to avoid inbreeding in allogamous plants that can be triggered before, during, or after pollination [1]. There are two SI systems differing mainly in their genetic control. Pollen compatibility determined by the diploid genotype of the pollen parent is referred to as sporophytic SI (SSI) [2]. In contrast, SI determined by the haploid pollen genotype is referred to as gametophytic SI (GSI) [3]. In the latter system, the male gamete does not display any dominance in diploid species because it is haploid. Thus, every allele is functional. SI can be controlled by either a single locus or few loci depending on the species. For example, in *Brassicaceae* and *Solanaceae,* SI is controlled by a single S gene, whereas for self-incompatible grasses, SI is controlled by a two-locus (S-Z) system. These two independent loci have among others been described for rye [4], perennial ryegrass [5], and Italian ryegrass [6]. The S-Z SI system results in low selfing rates and is functional even in polyploid grasses such as switchgrass, where tetraploid plants showed 0.35% self-compatibility, whereas octoploids reached 1.39% [7].

The proposed mechanism of SI in diploid grasses suggests that, if at least one allele at S or Z in the pollen differs from the recipient S-Z alleles, the pollen will be compatible with the stigma [2]. Fifty percent of the pollen produced by a genotype is compatible to pollen receptor genotype, If the pollen donor has one different allele at each S and Z, then the 75% of the pollen is compatible with the pollen receptor genotype [2]. If the pollen donor has one different allele at each S and Z, then the 75% of the pollen is compatible with the pollen receptor genotype. Finally, if the pollen donor has two different alleles at one of the two loci, then pollen is 100% compatible. The GSI system in diploid species with an S-Z system might lead to differences in reciprocal crosses [2].

**Figure 1:**
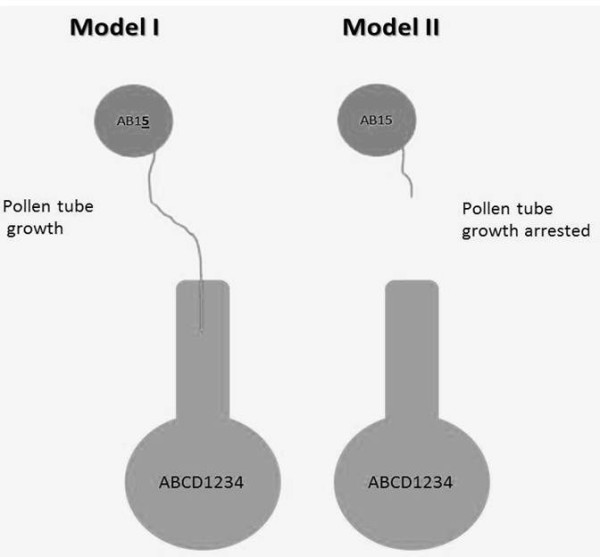
**Scheme of model differences for self-incompatible species with a S-Z system.** Model I: any different allele at either S or Z in the pollen different from S or Z on the pistil makes pollen is compatible. Model II: any S-Z allele combination present in the pollen grain that matches the pistil makes pollen incompatible.

In polyploid species the interaction between pollen and stigma alleles is more complicated due to the presence of more than two alleles at a locus. Throughout the manuscript, letters are used to identify alleles at S locus and numbers are used to identify alleles at Z. Here we propose two possible modes of interaction, named Model I and Model II. If any allele at either S or Z in the pollen differs from the alleles for S or Z on the pistil, pollen is compatible (Model I) [8]. If at least one allele at S and one allele at Z locus present in the pollen grain matches the pistil, then pollen is incompatible (Model II) [9,10]. For example pollen grain with genotype AB15 will be compatible under Model I because it has one different allele compared to the ABCD1234 female. In contrast, if Model II is true, pollen AB15 is incompatible with the female ABCD1234 because it shares one allele at S (A or B) and another at Z (1) (Figure 1). In addition to pollen-stigma interactions, the estimation of pollen compatibility percentages can be even more complex in polyploids due to inheritance patterns that can result in a high number of gametic haplotypes. Polyploidization is a common mechanism that occurred during evolution and it is the result of the fusion between unreduced gametes of different species (allopolyploidy) or of the same species (autotetraploidy). Genome analysis demonstrated that many plant genomes such as maize and soybean are ancient polyploids that went through diploidization events [11]. The nature of gamete fusion determines the behavior of chromosomes during meiosis. Allopolyploids show mostly preferential pairing of chromosomes in structures called bivalents (disomic), whereas autotetraploids pair with their respective homologous forming quadrivalentes (tetrasomic). In disomic inheritance there is no pairing between homeologues and, therefore, homologous chromosomes do not end up together in a gamete. Thus, for a given locus with genotype ABCD and preferential pairing between AB/CD, only four gametes (AC, BD, BC, and AD) are possible. In contrast, tetrasomic inheritance results in all six possible combinations for pairs of alleles (AB, AC, AD, BC, BD, and CD). Normal chromosome segregation implies that the two sister chromatids are distributed to different gametes. However, during quadrivalent formation in autotetraploids, crossing over between non-sister chromatids can occur. The probability that two sister chromatids end up in the same gamete is known as α. This gamete carries the alleles which are identical by descent [12] due to chromatid segregation and double reduction. For example, a plant with genotype ABCD for the S locus can produce AB, AC, AD, BC, BD, CD gametes under tetrasomic inheritance and AA, BB, CC and DD gametes if chromatid segregation and double reduction occur.

Consequently, it is possible in a tetraploid species to find gametes with alleles that are identical by descent (IBD), which results in a total of 10 possible different allele combinations in a gamete. When considering both SI loci, S and Z, respectively, 100 different gametes are possible. Thirty six gametes have the genotypes expected under meiosis with no crossing over, 48 are the result of crossing over between two non-sister chromatids and double reduction at either locus, and 16 gametes result from a double reduction at both loci. Chromatid segregation and double reduction increase the number of possible pollen genotypes and, therefore, increase the probability of cross-pollination between individuals with similar S and Z genotypes.

A software for numerical estimation and prediction of allelic and genotypic frequencies calculation for sporophytic SI systems has been published [13]. However, this model do not apply to gametophytic SI systems that do not display dominance. Segregation models for disomic and tetrasomic inheritance [14] as well as models for estimation of allele frequencies in polyploids [15] have also been published. However, this model lacks computerization of calculation. In addition, these models are generic for any locus, but do not deal with self-incompatibility or calculate pollen compatibility.

The program that we present here has the objective to support hypothesis testing under the different scenarios described above, especially for uncharacterized species. It can serve four different purposes: i) determine the number of progeny needed to differentiate between allo- and autotetraploidy; ii) obtain expected values of pollen compatibility for a pair-cross to maximize seed yield; iii) determine the SI mechanism (differentiate between Model I and II) in tetraploids; and iv) display information regarding S and Z distortion that can provide useful knowledge about segregation distortion of markers at these loci or linked markers for linkage mapping and population genetic studies.

## Implementation

The program was written in the C++ programming language using the freely available Qt compiler (Nokia, Germany) and compiled for the Windows™ operating system. A version for Mac OSX and Linux is under development.

### Calculator parameters for meiosis

The calculator is based on estimable meiotic parameters that influence chromosome segregation during meiosis of autotetraploids. For instance, parameter β determines, whether or not there is preferential pairing (disomic inheritance) between chromosomes. β is restricted to have values of 0 or 1. In case of tetrasomic inheritance or no preferential pairing β = 1. With four alleles per locus (i.e. ABCD) six gamete combinations are possible (AB, AC, AD, BC, BD, CD) under random pairing. In case of preferential pairing of homologues, β = 0. Furthermore, preferential pairing between genomes results in only four gamete combinations. To illustrate this further we named each genome *jklm* and alleles at a locus correspond to each genome in that order. For example, a genotype ABCD with preferential pairing between *jk/lm* has gametes with haplotypes AC, AD, BC, BD. In case of preferential pairing, only one out of the three possible pairing scenarios *jk/lm, jl/km,* or *jm/kl* can be true. This has been implemented in our calculations by defining a Δ value to test all three possible scenarios sequentially. Technically, if the Δ value is equal to 1 for one scenario, it is 0 for the other. Usually, the values for β and Δ do not differ for S and Z.

### Calculator frequencies estimation for segregation of S and Z

S and Z are independent loci. Therefore, normal chromosome segregation of a genotype with four different alleles for S or Z and with no preferential pairing between chromosomes *jk, jl, jm, kl, km,* and *lm* will produce six possible pollen haplotype combinations for each locus. Thus, the probability of a haplotype is 1/6. It is possible that recombination during multivalent formation in meiosis followed by double reduction can cause sister chromatids to segregate together during meiosis. Therefore, there is a chance of two identical alleles to end up in the same gamete by double reduction [12]. If we consider a single chromatid, only 1 out of the seven remaining is its corresponding sister chromatid. Therefore, the probability (α) of two sister chromatids to end up in the same gamete ranges from 0 to 1/7. The value of α is species-specific, and depends on the distance between the locus and the centromere [12,16]. For the *jklm* genotype, there are four possible gamete combinations that have two identical by descent alleles (IBD) i.e., *jj, kk, ll, mm*. Therefore, the frequency of each of these pollen genotypes is α/4. The closer S and Z are linked with the centromere, the smaller is the α value [16].

The input form of the program has a cell to enter α values to calculate gamete frequencies that can be different for each locus. For example, *Phalaris coerulescens* is a diploid species for which S and Z have been mapped. The S locus is located in a subcentromeric region of chromosome 1, and the Z locus is located on the long arm at the end of chromosome 2 [17]. In *P. coerulescens,* the α value for S will, therefore, be close to 0, whereas the one for Z should be larger. If the α value is unknown a default value such as 1/14 (average between the extremes of 0 and 1/7) could be used.

### Calculator input and settings

**Figure 2:**
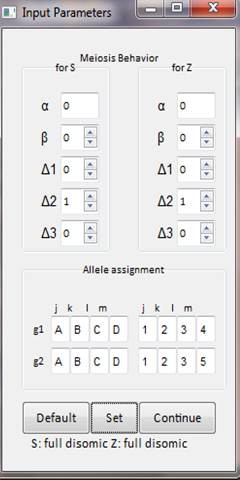
Input box for meiotic parameters for S-Z and genotype of two individuals.

The input window (Figure 2) has boxes for meiotic parameters for both loci (S and Z) as described above and for a given pair of genotypes. The default setting is disomic inheritance and preferential pairing between *jl/km* for both loci, which can be used as a null hypothesis. The parameters settings have some restrictions. For example, if β = 1, all Δ values are set to 0. If an α value is given, then β = 1. It is possible to enter the genotype for a pair of individuals. To our knowledge there is no information about allelic diversity for S and Z loci since the determinants have not been identified yet. However, we developed the software assuming that the maximum difference between two genotypes is eight different alleles at each locus. Therefore, the program accepts values for S defined by letters from A-H and numbers from 1-8 for Z. Settings will be summarized in words once input parameters are set. All the input settings will be summarized in words once input parameters are set.

### Calculator output

**Figure 3:**
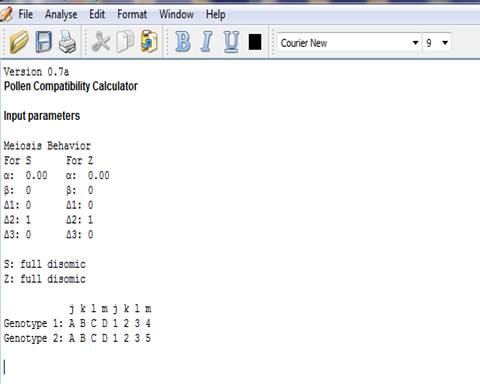
First output with input parameter summaries.

**Figure 4:**
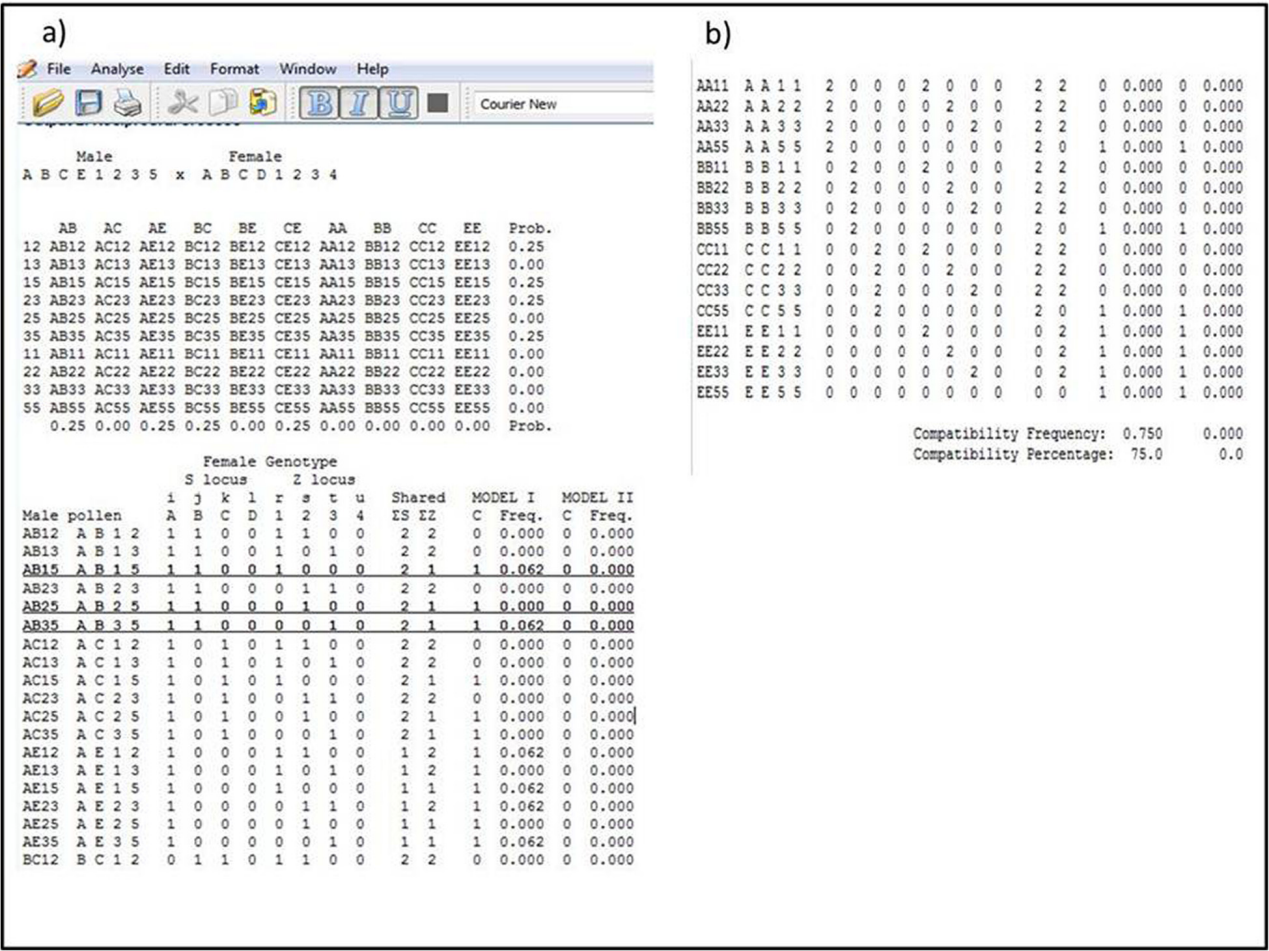
**a) Screenshot of pollen haplotype table and first 19 (out of 100) rows of pollen compatibility calculation for one of the two reciprocal crosses, underlined rows are examples of compatible pollen.b**) Last 16 (out of 100) pollen haplotypes compatibility and final compatibility percentage under two proposed models.

The initial output (Figure 3) obtained is a summary table of the input values. After the analysis is performed, the pollen haplotypes for each genotype and each locus are displayed. Pollen haplotypes are calculated based on the formulas in Table 1. Stift (2008) published formulas to calculate pollen haplotypes, which differ from ours in nomenclature. The second output contains tables for pollen genotypes and frequencies as well as pollen compatibility in reciprocal crosses between genotypes 1 and 2 (Figure 4a). For example for a genotype ABCD1234, the matrix for pollen compatibility has the 10X10 possible pollen haplotypes. Pollen haplotypes are arranged in column 1. In the column header of columns 2-5 and 6-9, the four female alleles for both, S and Z are displayed. Under each female allele, the numbers of identical alleles in the pollen are counted. Since pollen genotypes have only two alleles for each locus, the number of alleles it can possibly share with a female genotype at the S or Z locus, respectively, has to be 0, 1, or 2 alleles (see Additional file 1 for more description on input parameters and output).

**Table 1:** Formulas for pollen haplotype calculations

**Genotype**	**Equation**
**p(jj)**	1/4α
**p(kk)**	1/4α
**p(ll)**	1/4α
**p(mm)**	1/4α
**p(jk)**	1/6β-1/6α + (1-β) (1/4Δ2 + 1/4Δ3)
**p(jl)**	1/6β-1/6α + (1-β) (1/4Δ1 + 1/4Δ3)
**p(jm)**	1/6β-1/6α + (1-β) (1/4Δ1 + 1/4Δ2)
**p(kl)**	1/6β-1/6α + (1-β) (1/4Δ1 + 1/4Δ2)
**p(km)**	1/6β-1/6α + (1-β) (1/4Δ1 + 1/4Δ3)
**p(lm)**	1/6β-1/6α + (1-β) (1/4Δ2 + 1/4Δ3)

Genomes are named generically *jklm*. Therefore, alleles for S (i.e. ABCD) correspond to genomes *jklm* respectively; and alleles for Z (i.e. 1234) also correspond to genome *jklm* respectively. α is the probability of double reduction and ranges from 0- 0.14. β is a parameter for chromosome pairing during meiosis and can have values of 0 (disomic) or 1 (tetrasomic). Δ is a parameter that defines which chromosomes show preferential pairing.

For each possible pollen-stigma combination the number of shared alleles at the S and Z locus are given and summarized independently for S and Z. ΣS and ΣZ reflect the sum of shared alleles at S and Z, respectively (columns 10 and 11). In autotetraploids, for a given allele a locus can be monoallelic (AAAA), biallelic (AABB) or triallelic (AABC) [12]. Therefore, the program is designed to avoid double counting of alleles.

Pollen compatibility (C) is calculated for each pollen- stigma combination for the two models described above. Mathematically, the Model I algorithm states that if ΣS or ΣZ < 2, the pollen is compatible, therefore C = 1; otherwise C = 0. For Model II, the algorithm states that if ΣS or ΣZ = 0 then pollen is compatible and C = 1; otherwise C = 0. Thereafter, each C value is multiplied with the respective pollen haplotype frequency. The percentage of compatible pollen for a particular pollen-stigma genotype combination is the sum of the products between C values and pollen frequencies (Figure 4b).

**Figure 5:**
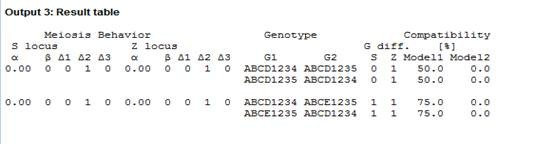
**Summary output of different runs (changes in genotypes).** First cross, genotypes have one different allele at Z and a pollen compatibility of 50% under Model I and 0% under Model II. In the following case, genotypes differ in one allele at S and one at Z and pollen compatibility increase 25% under Model I and remains 0% under Model II. Meiotic parameters are kept constant for S and Z.

Our program allows the user to continue the analysis with similar meiotic parameters. Thus, the final output (Figure 5) is a summary table containing meiotic parameters used, genotype of individuals and the compatibility percentages under both models.

## Results and discussion

### Difference between preferential and non-preferential pairing

A male with genotype ABCD1234 will produce gametes with different haplotypes depending on the mode of pairing during meiosis. Tables 2 &3 show possible pollen haplotypes, where shaded pollen haplotypes were produced under the assigned pairing and meiotic parameters. If such a male is crossed onto a female with different alleles at S and Z (i.e., EFGH5678), it is possible to determine the meiotic behavior based on the progeny. Based on output information, it is also possible to determine the number of progeny needed, to screen to find a certain haplotype.

**Table 2:** Pollen haplotypes and their respective frequencies for a male ABCD1234 with disomic inheritance

Disomic inheritance: β = 0 **Δ1 = 1**							
		**0**	**0.25**	**0.25**	**0.25**	**0.25**	**0**
		**AB**	**AC**	**AD**	**BC**	**BD**	**CD**
**0**	**12**	AB12	AC12	AD12	BC12	BD12	CD12
**0.25**	**13**	AB13	**AC13**	**AD13**	**BC13**	**BD13**	CD13
**0.25**	**14**	AB14	**AC14**	**AD14**	**BC14**	**BD14**	CD14
**0.25**	**23**	AB23	**AC23**	**AD23**	**BC23**	**BD23**	CD23
**0.25**	**24**	AB24	**AC24**	**AD24**	**BC24**	**BD24**	CD24
**0**	**34**	AB34	AC34	AD34	BC34	BD34	CD34
Disomic inheritance: β = 0 Δ2 = 1							
		**0.25**	**0**	**0.25**	**0.25**	**0**	**0.25**
		**AB**	**AC**	**AD**	**BC**	**BD**	**CD**
**0.25**	**12**	**AB12**	AC12	**AD12**	**BC12**	BD12	**CD12**
**0**	**13**	AB13	AC13	AD13	BC13	BD13	CD13
**0.25**	**14**	**AB14**	AC14	**AD14**	**BC14**	BD14	**CD14**
**0.25**	**23**	**AB23**	AC23	**AD23**	**BC23**	BD23	CD23
**0**	**24**	AB24	AC24	AD24	BC24	BD24	CD24
**0.25**	**34**	**AB34**	AC34	**AD34**	**BC34**	BD34	**CD34**
Disomic inheritance: β = 0 Δ3 = 1							
		**0.25**	**0.25**	**0**	**0**	**0.25**	**0.25**
		**AB**	**AC**	**AD**	**BC**	**BD**	**CD**
**0.25**	**12**	**AB12**	**AC12**	AD12	BC12	**BD12**	**CD12**
**0.25**	**13**	**AB13**	**AC13**	AD13	BC13	**BD13**	**CD13**
**0**	**14**	AB14	AC14	AD14	BC14	BD14	CD14
**0**	**23**	AB23	AC23	AD23	BC23	BD23	CD23
**0.25**	**24**	**AB24**	**AC24**	AD24	BC24	**BD24**	**CD24**
**0.25**	**34**	**AB34**	**AC34**	AD34	BC34	**BD34**	**CD34**

Possible pollen haplotypes produced under the designed preferential pairing are in bold (Δ1-3).

**Table 3:** Pollen haplotypes and their respective frequencies for a male ABCD1234 with tetrasomic inheritance

**Tetrasomic inheritance: β = 1 all Δ = 0 α = 0**											
		**0.17**	**0.17**	**0.17**	**0.17**	**0.17**	**0.17**				
		**AB**	**AC**	**AD**	**BC**	**BD**	**CD**				
**0.17**	**12**	AB12	AC12	AD12	BC12	BD12	CD12				
**0.17**	**13**	AB13	AC13	AD13	BC13	BD13	CD13				
**0.17**	**14**	AB14	AC14	AD14	BC14	BD14	CD14				
**0.17**	**23**	AB23	AC23	AD23	BC23	BD23	CD23				
**0.17**	**24**	AB24	AC24	AD24	BC24	BD24	CD24				
**0.17**	**34**	AB34	AC34	AD34	BC34	BD34	CD34				
**Tetrasomic Inheritance + Chromatid Segregation + Double Reduction β = 1 all Δ = 0 α = 0.14**											
		**0.14**	**0.14**	**0.14**	**0.14**	**0.14**	**0.14**	**0.04**	**0.04**	**0.04**	**0.04**
		**AB**	**AC**	**AD**	**BC**	**BD**	**CD**	**AA**	**BB**	**CC**	**DD**
**0.17**	**12**	AB12	AC12	AD12	BC12	BD12	**CD12**	**AA12**	**BB12**	**CC12**	**DD12**
**0.17**	**13**	AB13	AC13	AD13	BC13	BD13	**CD13**	**AA13**	**BB13**	**CC13**	**DD13**
**0.17**	**14**	AB14	AC14	AD14	BC14	BD14	**CD14**	**AA14**	**BB14**	**CC14**	**DD14**
**0.17**	**23**	AB23	AC23	AD23	BC23	BD23	**CD23**	**AA23**	**BB23**	**CC23**	**DD24**
**0.17**	**24**	AB24	AC24	AD24	BC24	BD24	**CD24**	**AA24**	**BB24**	**CC24**	**DD24**
**0.17**	**34**	AB34	AC34	AD34	BC34	BD34	**CD34**	**AA34**	**BB34**	**CC34**	**DD34**
**0.04**	**11**	**AB11**	**AC11**	**AD11**	**BC11**	**BD11**	*CD11*	*AA11*	*BB11*	*CC11*	*DD11*
**0.04**	**22**	**AB22**	**AC22**	**AD22**	**BC22**	**BD22**	*CD22*	*AA22*	*BB22*	*CC22*	*DD22*
**0.04**	**33**	**AB33**	**AC33**	**AD33**	**BC33**	**BD33**	*CD33*	*AA33*	*BB33*	*CC33*	*DD33*
**0.04**	**44**	**AB44**	**AC44**	**AD44**	**BC44**	**BD44**	*CD44*	*AA44*	*BB44*	*CC44*	*DD44*

Pure tetrasomic inheritance produces all 36 possible genotypes are shown in regular format. Haplotypes in bold are the ones formed by chromatid segregation and haplotypes in italic are formed by double reduction. The probabilities of each S or Z allele combination are given; the gamete frequency can be calculated as joined probability of the frequency at each locus.

Assuming that markers are available to distinguish alleles for S and Z, the stepwise procedure to determine meiotic behavior for S and Z loci would be as follows: first, distinguish between disomic and tetrasomic inheritance. If disomic inheritance occurs, under any type of preferential pairing only 16 pollen haplotypes are possible. In contrast, if tetrasomic inheritance occurs, 36 pollen haplotypes are possible. To distinguish between both, it is necessary to evaluate the presence or absence of any of the 20 (36-16) haplotypes, that are indicative of tetrasomic inheritance.

If disomic inheritance is true, it can be determined, which genomes are pairing during meiosis. One haplotype that distinguishes between *jk/lm, jl/km* or *jm/kl* needs to be identified. In the example presented in Table 2, AB12 is a haplotype that can differentiate between *jk/lm* and *jl/km*. The probability of occurrence of these haplotypes in the progeny is 0.0625, to find at least one progeny we use the formula described by Sedcole (method III) [18]: carrying a specific pollen haplotype,

(1) $$n=\frac{\left[2\left(r-0.5\right)+z^{2}\left(1-q\right)\right]+z{\left[z^{2}{\left(1-q\right)}^{2}+4\left(1-q\right)\left(r-0.5\right)\right]}^{\frac{1}{2}}}{2q}$$

Where n = total number of plants to be screened

r- required number of plants with the desired genotype

q = frequency of plants with the desired genotype

p = probability of recovering the required number of plants with the desired genotype

z- probability based on a z distribution

Therefore, we need to screen at least 56 plants in this example to be 95% confident to find one offspring carrying the AB12 haplotype. If Δ2 is true, it is necessary to select a haplotype to distinguish it from Δ3 (i.e., AD12). Using the same calculation as above, 56 individuals need to be screened to find at least one genotype carrying the AD12 haplotype with a 95% probability.

On the other hand, if tetrasomic inheritance is true for our species, it is necessary to determine, whether chromosome segregation or double reduction occurs. The probability of finding one haplotype produced by chromatid segregation is 0.2688 (the sum of the frequencies of haplotypes of light shaded boxes in Table 3). Using the same formula as above, to find at least one of these haplotypes in the progeny with a 95% confidence, a total of 11 individuals need to be analyzed. Furthermore, if we want to assess the occurrence of double reduction, which occurs with a frequency of 0.0256 (the sum of the frequencies of no shaded lines in Table 3), a total of 139 individuals need to be screened.

### Model testing

**Figure 6:**
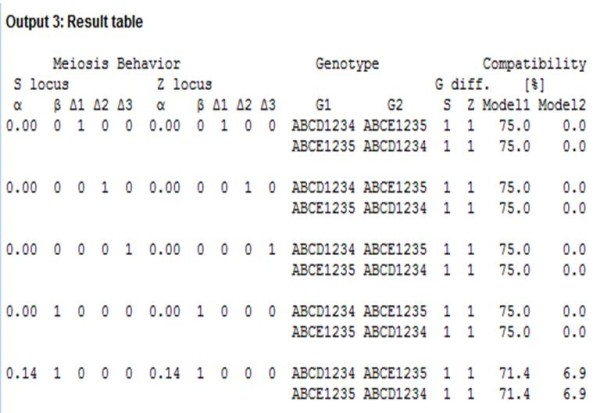
**Program output 3.** Summary output that show differences in pollen compatibilities between Model I and II, and disomic or tetrasomic inheritance.

**Figure 7:**
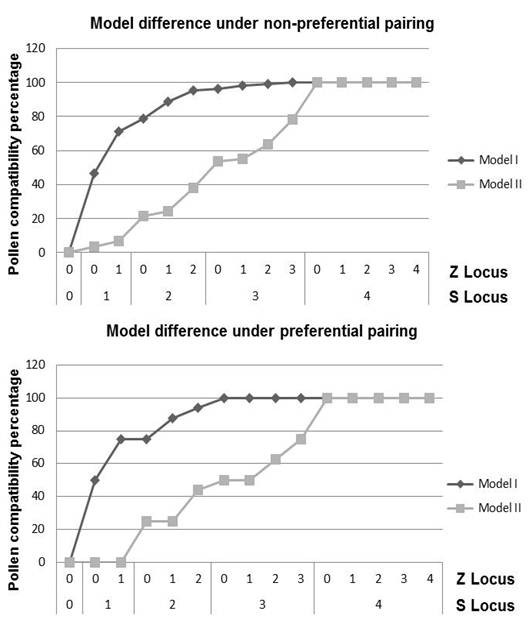
**Hypothesis testing of SI models.** Analysis of differences in pollen compatibility under Model I and Model II for species under non-preferential pairing. Expected pollen compatibility percentages are plotted against the number of different alleles at S and Z loci.

Our program can be used to determine the most likely model underlying pollen compatibility. For example, in a cross between ABCD1234 and ABCE1235, these genotypes have one different allele at each locus. Under Model I, reciprocal crosses between these genotypes reaches 75% pollen compatibility. In contrast, under Model II, this cross is completely incompatible. Multivalent formation, chromatid segregation and double reduction during meiosis have an effect on pollen compatibility. A similar cross, in an autotetraploid species (β = 1) with chromatid segregation and double reduction (α = 1/7), will have 71.6% pollen compatibility under Model I or 6.9% under Model II (Figure 6). In general, Model II is more restrictive than Model I. Model I ranges from 0% to 50% compatibility, when one different allele is present at either S or Z. In contrast, Model II has values lower than 50%, if there are fewer than three different alleles at any locus (Figure 7).

### Allele composition

If meiotic parameters are known or can be calculated, but no markers are available for genotyping S and Z, it is possible to infer the number of alleles segregating in a population of a pair cross. In a plant species with tetrasomic inheritance (β = 1) and chromatid segregation/double reduction (α = 1/7), the number of segregating alleles between the pollen donor and female plays an important role on the pollen compatibility in reciprocal crosses. For instance, if the number of total segregating alleles is four or more for at least one locus (i.e., ABCD1234 X AAAB1115), pollen compatibility is higher than 90% under Model I but only 63% for Model II (Table 4). However, the distribution of these alleles in the two genotypes is important for the pollen compatibility in reciprocal crosses. If ABCD1234 is used as the pollen donor it has two and three alleles for S and Z, respectively, which differ from the female AAAB1115. In contrast, when AAAB1115 is used as the pollen donor, it has zero and one allele for S and Z, respectively, that differ from the female ABCD1234, resulting in a pollen compatibility of 47% under Model I, or 4% under model II (Table 3). In addition, if the number of segregating alleles between genotypes drops to two, pollen compatibility is significantly reduced. For instance, a cross between AAAB1115 X AABB1111 will result in no pollen compatibility under any Model. However, if AABB1115 is used as male the single different allele at Z will cause an increase of pollen compatibility to 47% under Model I, or 4% under Model II (Table 4).

**Table 4:** Differences in reciprocal crosses between genotypes that have alleles identical by descent (IBD)

		**Total segregating alleles**		**Pollen donor alleles differing from female**		**Female alleles differing from pollen donor**		**Pollen**	**compatibility**
**Pollen Donor**	**Female**	**S**	**Z**	**S**	**Z**	**S**	**Z**	**Model I**	**Model II**
ABCD1234	**AAA**B**111**5	4	5	2	3	0	1	99.3	63.4
**AAAB1115**	ABCD1234	4	5	0	1	2	3	46.5	3.5
**AAAD1234**	**AAA**B**111**5	3	5	1	3	1	1	98.1	55.1
**AAAB1115**	**AAA**D1234	3	5	1	1	1	3	71.4	6.9
**AABD1134**	**AAA**B1115	3	4	1	2	0	1	88.6	24.1
**AAAB1115**	**AA**BD**11**34	3	4	0	1	1	2	46.5	3.5
**AABD1114**	**AAA**B**111**5	3	3	1	1	0	1	71.4	6.9
**AAAB1115**	**AA**BD**111**4	3	3	0	1	1	1	46.5	3.5
**AABB1111**	**AAA**B**111**5	2	2	0	0	0	1	0	0
**AAAB1115**	**AABB1111**	2	2	0	1	0	0	46.5	3.5

Alleles that are IBD are in bold and underlined, First column (total number of segregating alleles) is the number of different alleles between S or Z across genotypes. The columns that follow count the number of different alleles between males and females.

The information provided by PollenCALC can be used to design experiments in order to test hypotheses related to the pairing and segregation of chromosomes during meiosis in tetraploid species with a gametophytic S-Z SI system. The program can be used if linked or functional markers for S and Z are available or not.

The genetic mechanism of SI is still unknown for some self-incompatible tetraploid grasses. Also, there are no markers available for S and Z which makes it difficult to understand and predict pollen compatibility. As described above, there are two possible models for pollen compatibility. *In vitro* or *in vivo* pollinations can be performed to determine, which of the two models most likely applies. As demonstrated, only few different alleles are required for Model I. Furthermore, under Model I, if only one different allele is found at either S or Z, pollen compatibility reaches 50%, whereas for Model II values below 50% are common when the number of different alleles is below three for any locus. “Both *in vitro* and *in vivo* pollination test provide bias estimates of pollen compatibility. For instance, current *in vitro* pollination tests rely on imaging and counting to provide estimates of pollen compatibility. *In vivo* pollination analyzed as seed set can be affected by pollen death due to environmental condition and pre or post zygotic abortion. Nevertheless, PollenCALC can be used to design experiments in order to identify genotypes that are most informative to distinguish the two models i.e. genotypes with extreme pollen compatibilities. Eventually the use of S and Z linked markers are required for confirmation.”

There are other applications for this program when markers are available. For example, genome-wide markers are used in several studies to determine preferential pairing in polyploids. In species with SI, segregation of markers linked or in linkage disequilibrium with S and Z are distorted, which can result in biased interpretation of marker segregation data [19,20]. PollenCALC can help to identify genotypes that can distinguish between types of pairing. Knowledge about pairing and inheritance in a given species makes it possible to correct mapping distances for segregation distortion due to SI in mapping experiments.

Also, PollenCALC calculates pollen haplotype frequencies of individuals in pair crosses and can be used to calculate pollen compatibility between those genotypes in reciprocal crosses. This information can also be used to maximize seed yield in hybrid breeding programs [21]. If markers for S and Z are available, they can be used in combination with our software in breeding programs. For instance in a hybrid seed production programs, PollenCALC can be used to select genotypes with specific S-Z genotypes within one heterotic group that contrast with the S-Z genotype from the individuals in the other heterotic group. If alleles are different between groups, seed yield will be maximized. Also, it is possible to determine potential progeny genotype and frequencies by combining haplotypes and frequencies. In the longer run, this program can be used to simulate allele and genotypes frequencies for S, Z, and loci genetically linked to S and Z over multiple generations. Thus, PollenCALC is a useful tool for plant geneticists and plant breeders working with tetraploid species.

## Conclusion

PollenCALC is the only program that we are aware of that is able to calculate gamete haplotypes and pollen compatibility for tetraploid species with a gametophytic S-Z SI. The frequencies obtained can be used to determine the number of individuals needed to test different models for GSI, to optimize experimental designs, and to take segregation distortion of linked markers into account, indicating linkage disequilibrium with SI loci.

## Availability and requirements

Project name: PollenCALC

Project home page: https://github.com/BerndWollenweber/PollenCALC/downloads

Operating system(s): tested under Windows XP, Windows Vista, Windows 7

Programming language: C++

Other requirements: none.

License: free non-commercial research-use license.

Any restrictions to use by non-academics: none.

## Abbreviations

SI: Self-incompatibility; GSI: Gametophytic self-incompatibility; SSI: Sporophytic self-incompatibility.

## Competing interests

The authors declare that they have no competing interests.

## Authors’ contributions

AAA has developed the algorithms and carried out initial design for the calculation and estimation of pollen compatibility under the two proposed models. BW conceived the software, carried out its design and participated in writing the draft of the manuscript. UKS and TL have participated in writing the draft of the manuscript. All authors have read and approved the final manuscript.

## Supplementary Material

Additional file 1 Pollen compatibility calculator USER MANUAL. Click here for file
