# Supplementary material for: PollenCALC: Software for estimation of pollen compatibility of self-incompatible allo- and autotetraploid species
Source: BMC Bioinformatics. 2012 Jun 7;13:125. doi: 10.1186/1471-2105-13-125 (PMC3439690; doi:10.1186/1471-2105-13-125)
Supplement: Additional file 1 — Pollen compatibility calculator USER MANUAL. [file 1471-2105-13-125-S1.doc]

Pollen compatibility calculator
USER MANUAL

BACKGROUND

Each locus in a tetraploid has four alleles, for example ABCD.

Mathematically, each allele can pair with each other and itself. As a result, ten possible haplotypes (pair of alleles) are possible (i.e. AA, BB, CC, DD, AB, AC, AD, BC, BD, CD).

Biologically, the probability of occurrence of these combinations depends on different events that happen during gamete formation (Meiosis).

INTRODUCTION - Chromosome pairing and description of parameters

Generically, each genome is named as jklm. If the multiple genomes have the same origin, chromosomes can pair at random with each other. β (beta) determines preferential pairing. If there is no preferential pairing, β=1 and six gamete haplotypes are possible (AB, AC, AD, BC, BD, CD) - each with a frequency of 1/6.

If there is preferential pairing, β=0. Preferential pairing can occur between different genomes. For example, if pairing occurs between jk/lm, alleles that are in jk or lm chromosomes cannot end up in the same gamete. Therefore, it is necessary to assign a Δ (delta) value to determine which allele combinations are possible in gametes.

Preferential pairing between jk/lm, is defined by setting an indicator variable Δ1=1, and indicator variables Δ2 and Δ3=0. Similarly, in case of jl/km Δ2=1, while Δ1 and Δ3=0. For jm/kl, Δ1 and Δ2 = 0, and Δ3 = 1.

Finally, α (alpha) is a parameter that determines the probability of two alleles to end up in the same gamete. For example, an ABCD genotype can produce (AB, AC, AD, BC, BD, CD) as described above and also (AA, BB, CC, DD), the latter gametes each at equal frequency.

All these values can be expressed as equations:

| **Genotype Prob** | **Equations** |
| --- | --- |
| p(jj) | 1/4α |
| p(kk) | 1/4α |
| p(ll) | 1/4α |
| p(mm) | 1/4α |
| p(jk) | 1/6β-1/6α +(1-β) (1/4Δ2+1/4Δ3) |
| p(jl) | 1/6β-1/6α +(1-β) (1/4Δ1+1/4Δ3) |
| p(jm) | 1/6β-1/6α +(1-β) (1/4Δ1+1/4Δ2) |
| p(kl) | 1/6β-1/6α +(1-β) (1/4Δ1+1/4Δ2) |
| p(km) | 1/6β-1/6α +(1-β) (1/4Δ1+1/4Δ3) |
| P(lm) | 1/6β-1/6α +(1-β) (1/4Δ2+1/4Δ3) |

PROGRAM DESCRIPTION

Start the program by clicking **Analyse/Input** (Shortcut CTRL A) on the menu. The input box appears.


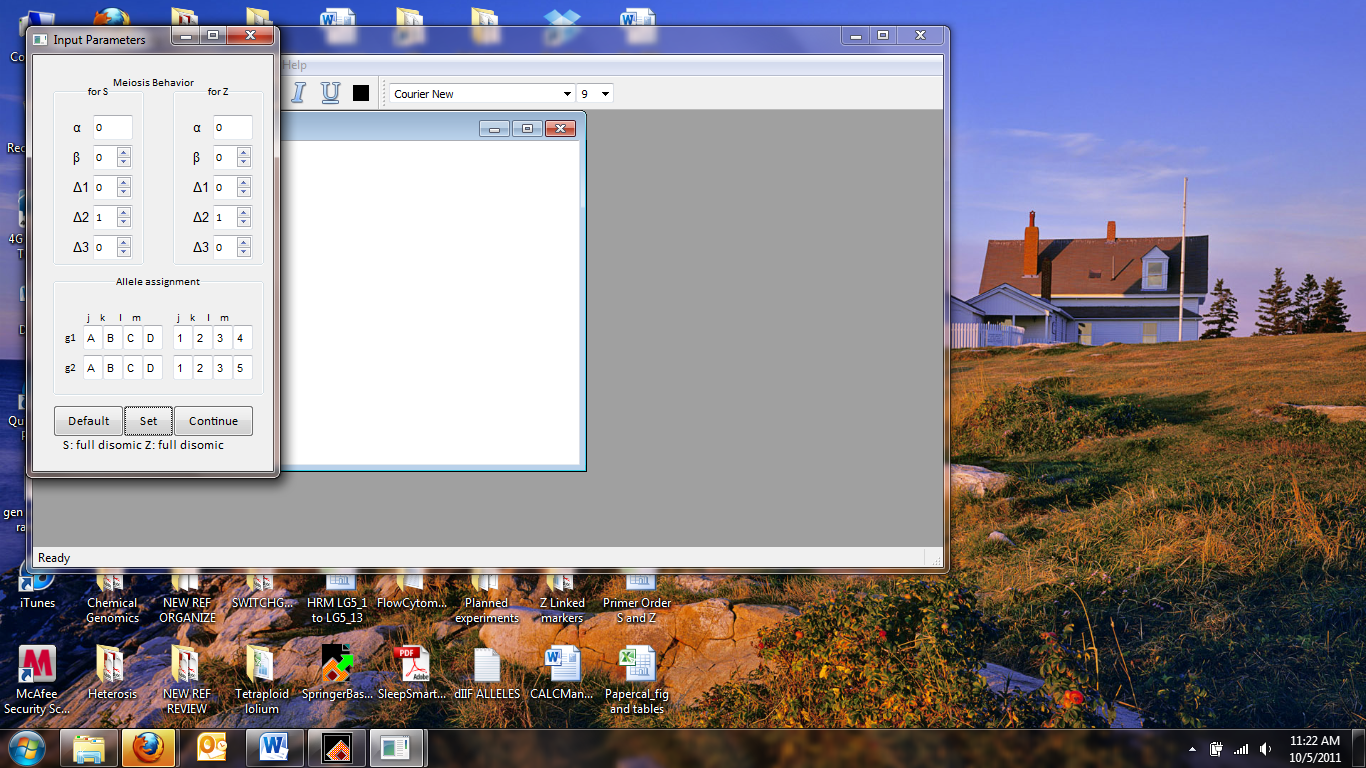


Meiotic parameters (α, β, and Δ) can be set according to meiotic behavior, if known for the species. Otherwise, different settings can be used for hypothesis testing.

These parameters have certain restrictions:

- α values range from 0 to 1/7 and can be >0 only if β=1
- β can have values of 1 or 0, as described above.
- Δ can have values of 1 or 0; the sum of Δ values within a locus has to be 1.

Also in the input box, there are boxes for S and Z genotypes for two individuals (g1 and g2)

Alleles for S must be introduced as capital letters A-I and alleles for Z must be introduce as numbers from 1-9.

Below the genotype specifications, are 3 action buttons:

- **Default**: sets meiotic parameters α=0 and β=0 and Δ_2_ =1, all other values are 0
- **Set**: This button must be pressed after all parameters have been entered. A description of the resulting meiotic behavior appears at the bottom of the input window.
- **Continue**: this button stores and transfers the parameters to the output window

Output:

The first output is a summary of the input parameters as shown in the following screenshot


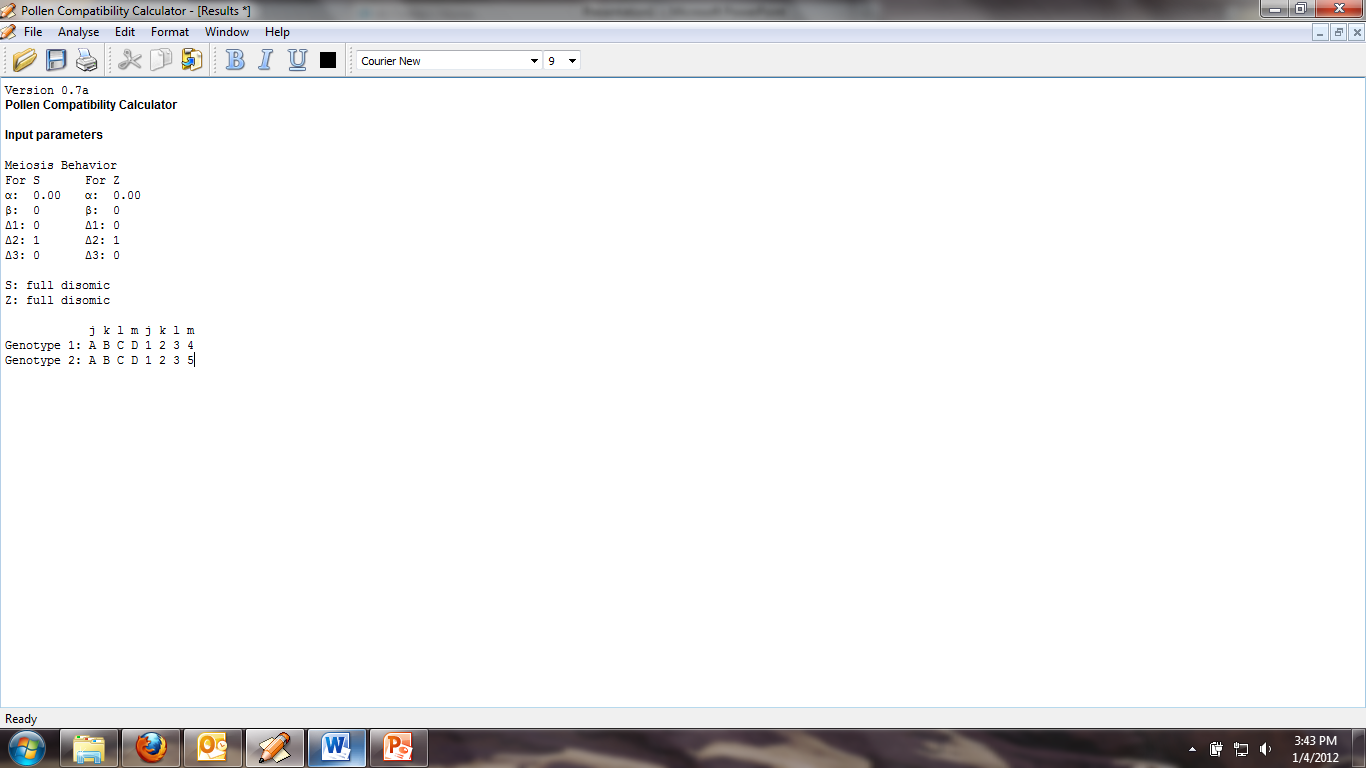


To get the output, hit the Analyze tab and click on **Analyse/Output1**. (Shortcut CTRL R).

This will display the first output with haplotype frequencies and probabilities for S and Z or both individuals


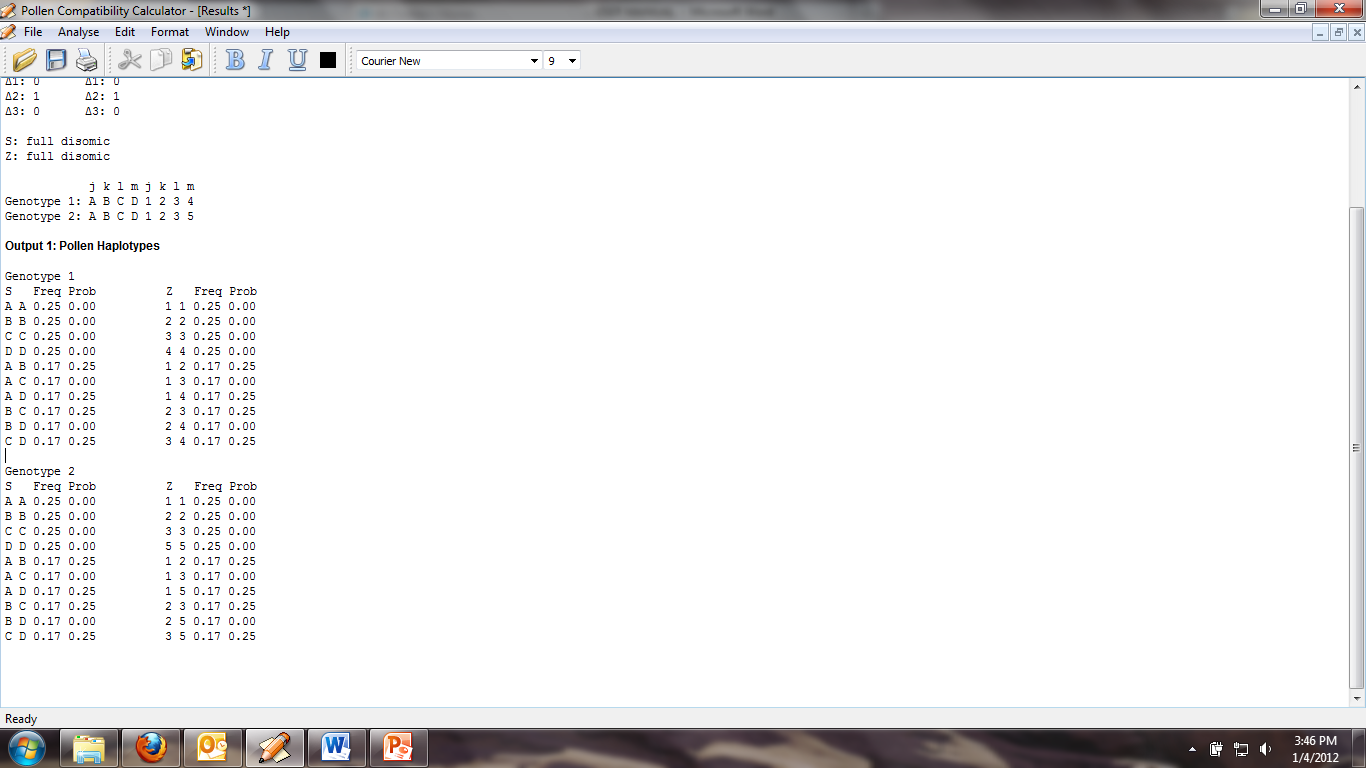


To get combined gametes and pollen compatibility calculation, **click Analyze/Output2** (shortcut CTRL T). This will display first the directional cross that is being calculated. Then a table with combined pollen haplotypes for S and Z is displayed.


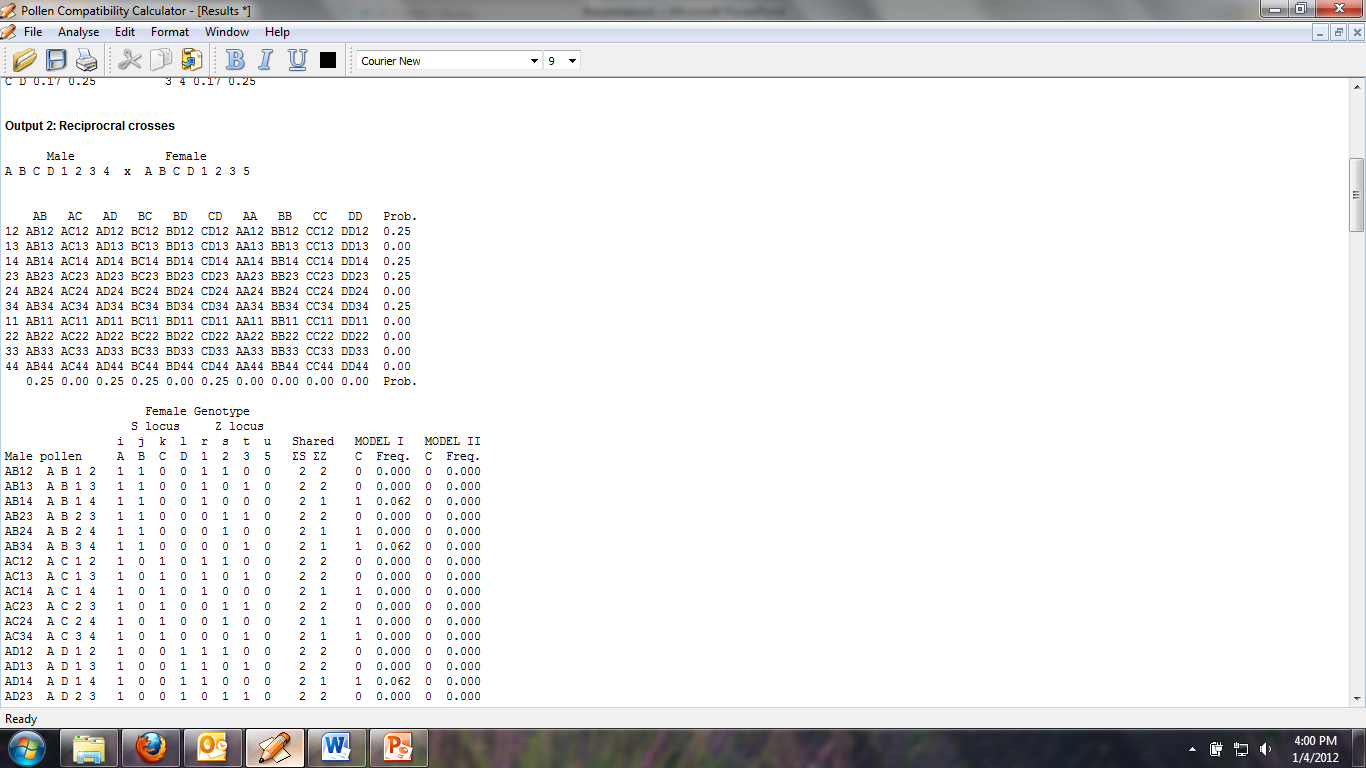


Also, a matrix for pollen compatibility is displayed in which each allele present in the pollen is compared to the alleles present in the female. If the allele is present in both, a number (1) is added to the corresponding cell. If the allele is not shared between pollen haplotype and female, then a number (0) is added. Then, the number of shared alleles at S and Z are summed up under the column ΣS and ΣZ, respectively.

Finally, in the column assigned as (C) compatibility is determined under a given model.

For Model I if ΣS or Σz < 2, C=1. This means that pollen is compatible; otherwise (0) means that pollen is incompatible. In contrast, for Model I if ΣS or ΣZ = 0, C=1. This means that pollen is compatible; otherwise (0) means that pollen is incompatible. The resulting binomial matrix is multiplied by the corresponding pollen frequency calculated based on meiotic parameters.


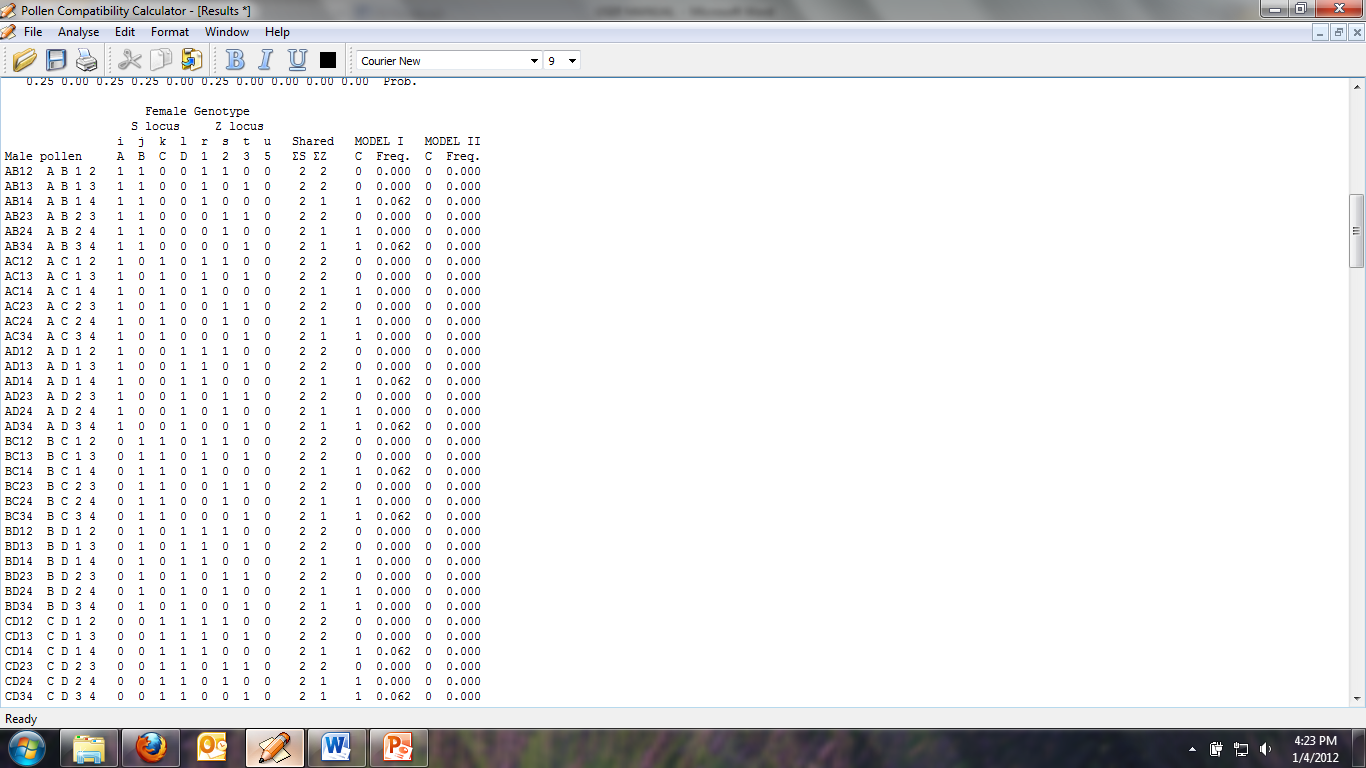


At the end of the matrix the pollen frequencies are summed to determine the total pollen compatibility under each model as shown in the following screenshot

Model I Model II


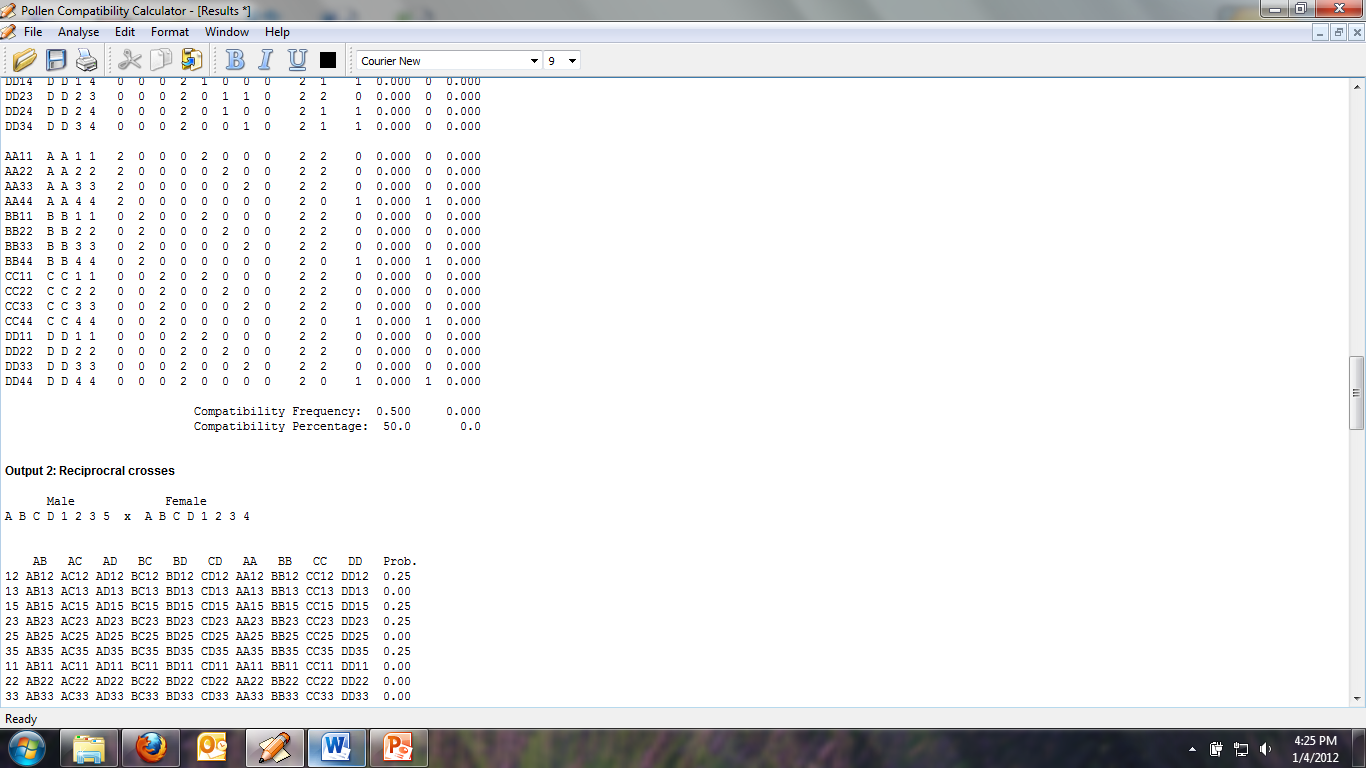


The same is done for the reciprocal cross.

At the end of the analysis the following box appears:


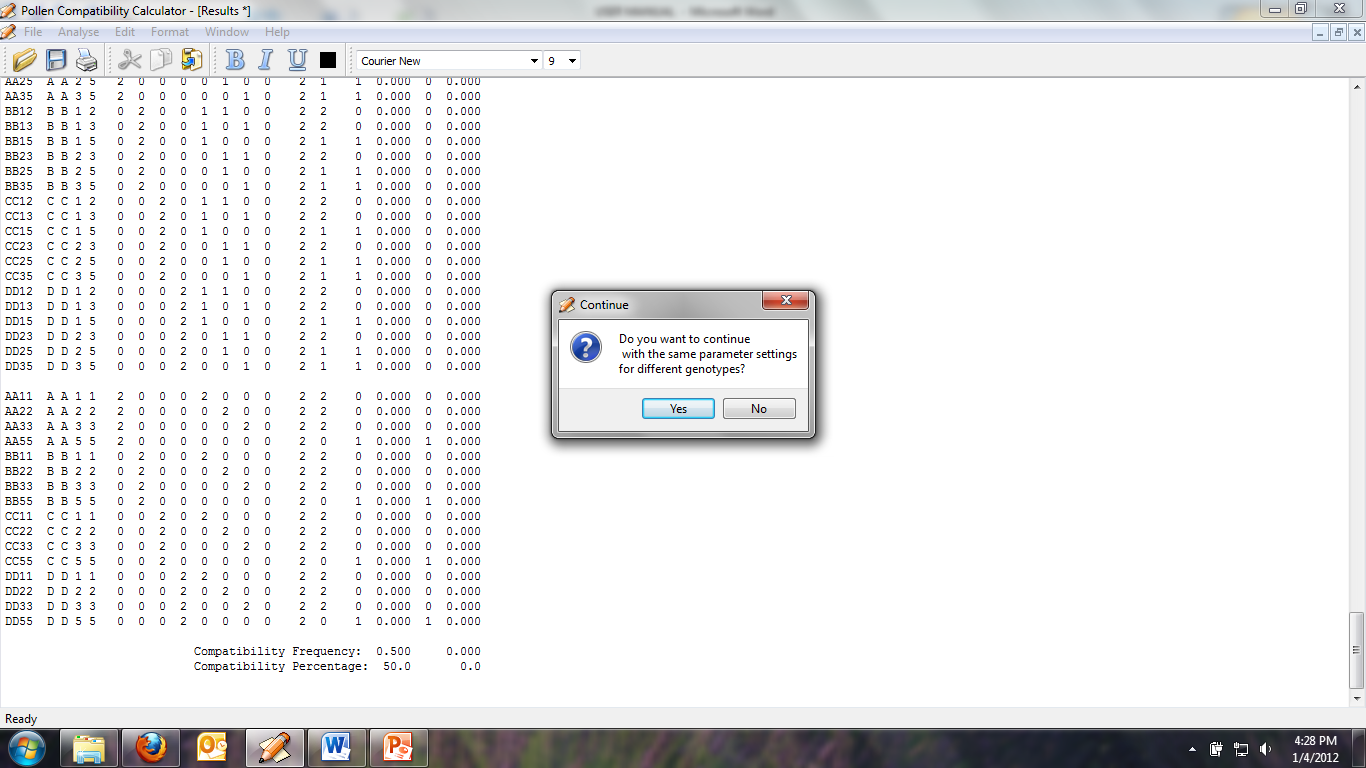


If “yes: is selected, the input box reappears and changes can be made in all cells. This allows

serial analysis with small differences in the settings. Once all the desired changes and the resulting analyses are done, clicking the “no” option results in the output of the summary table. The following screenshot shows an example:


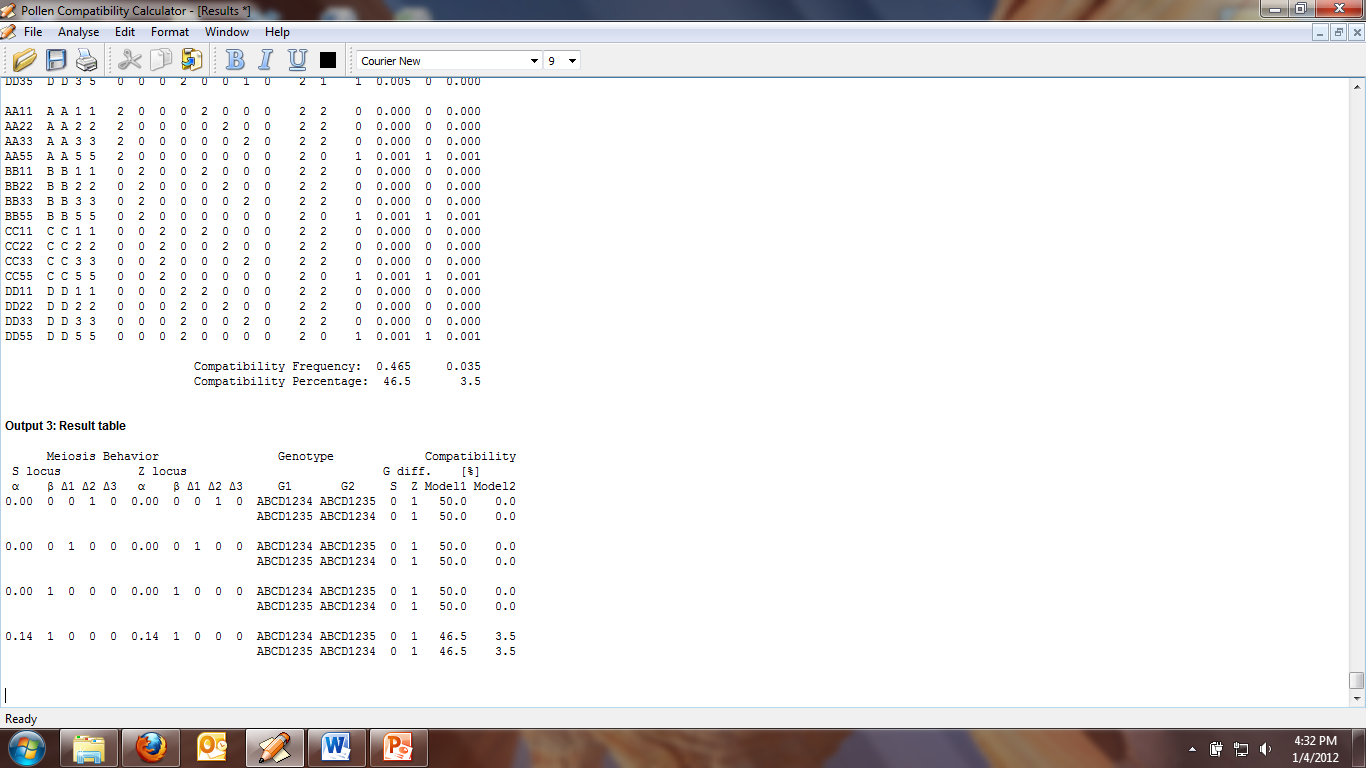


The text in the output window can be formatted (bold, italic, underlined, color, font-type, font-height) either via the menu or the icons in the icon-bar on top of the output window. Highlighted text can be cut, copied and pasted in the same manner. The text in the output window can be saved by clicking on the relevant icons in the icon-bar or the menu.

You don’t have to close the output window to start another run of the program. Just click on Analyse/Input again to start another set of analyses as described above.
